# KCNE1 and KCNE3 β-Subunits Regulate Membrane Surface Expression of Kv12.2 K^+^ Channels *In Vitro* and Form a Tripartite Complex *In Vivo*

**DOI:** 10.1371/journal.pone.0006330

**Published:** 2009-07-22

**Authors:** Sinead M. Clancy, Bihan Chen, Federica Bertaso, Julien Mamet, Timothy Jegla

**Affiliations:** Department of Cell Biology, Institute for Childhood and Neglected Diseases, The Scripps Research Institute, La Jolla, California, United States of America; University of Virginia, United States of America

## Abstract

Voltage-gated potassium channels that activate near the neuronal resting membrane potential are important regulators of excitation in the nervous system, but their functional diversity is still not well understood. For instance, Kv12.2 (ELK2, KCNH3) channels are highly expressed in the cerebral cortex and hippocampus, and although they are most likely to contribute to resting potassium conductance, surprisingly little is known about their function or regulation. Here we demonstrate that the auxiliary MinK (KCNE1) and MiRP2 (KCNE3) proteins are important regulators of Kv12.2 channel function. Reduction of endogenous KCNE1 or KCNE3 expression by siRNA silencing, significantly increased macroscopic Kv12.2 currents in *Xenopus* oocytes by around 4-fold. Interestingly, an almost 9-fold increase in Kv12.2 currents was observed with the dual injection of KCNE1 and KCNE3 siRNA, suggesting an additive effect. Consistent with these findings, over-expression of KCNE1 and/or KCNE3 suppressed Kv12.2 currents. Membrane surface biotinylation assays showed that surface expression of Kv12.2 was significantly increased by KCNE1 and KCNE3 siRNA, whereas total protein expression of Kv12.2 was not affected. KCNE1 and KCNE3 siRNA shifted the voltages for half-maximal activation to more hyperpolarized voltages, indicating that KCNE1 and KCNE3 may also inhibit activation gating of Kv12.2. Native co-immunoprecipitation assays from mouse brain membranes imply that KCNE1 and KCNE3 interact with Kv12.2 simultaneously *in vivo*, suggesting the existence of novel KCNE1-KCNE3-Kv12.2 channel tripartite complexes. Together these data indicate that KCNE1 and KCNE3 interact directly with Kv12.2 channels to regulate channel membrane trafficking.

## Introduction

Neuronal voltage-gated (Kv) potassium channels that open at membrane potentials close to the threshold for action potential generation have a profound effect on neuronal excitability because of their ability to stabilize membrane potentials below the threshold for action potential initiation. Kv12 (Elk) channels are among the most interesting sub-threshold Kv channels because they activate at more hyperpolarized potentials than other outwardly rectifying Kv channels [1]–[5], suggesting a strong influence on the resting membrane potential. Three distinct mammalian Kv12 channel genes have been identified: Kv12.1, Kv12.2 and Kv12.3 [1], [2], [6]; all three are primarily expressed in the brain [2], [5], [7], [8]. However, *in situ* hybridization and real time RT-PCR studies have revealed that Kv12.2 is the most abundant, with high expression evident in the dentate gyrus, hippocampal pyramidal neurons, cortex, cerebellum and basal ganglia [5], [8]. Interestingly these regions of the brain have been associated with pathophysiological hyperexcitability; disruption of K^+^ currents in the dentate gyrus can lead to abnormal hippocampal synchronization and temporal lobe seizures [9]. However, despite the strong potential for Kv12 family K^+^ channels as important regulators of neuronal function and cellular excitability, the cellular neurophysiology, fundamental regulation mechanisms and molecular composition of these channels remains unknown.

We reasoned that we could gain insights into the regulation and molecular composition of Kv12 channels by identifying interacting proteins. Interestingly, previous studies have demonstrated that the single transmembrane domain β-subunits MinK and MinK-related peptides (MiRPs), which are encoded by the KCNE genes, modify and often radically alter gating, conductance and the pharmacology of a diverse range of Kv channels [10]. For example KCNE β-subunits alter KCNQ, ERG, and HCN channel currents, as well as members of the Kv1-4 channel family [11]–[17]. As Kv12.2 channels are closely related to ERG channels [5], [18], we hypothesized that endogenous KCNE genes similarly regulate Kv12.2 channel function.

Historically the role of KCNE genes in Kv channel regulation has primarily focused on mammalian heart; with more recent studies focusing on the gastrointestinal tract and skeletal muscle [15], [19]–[23]. To date five known members of the KCNE gene family have been identified (KCNE1-5), and all have been shown to effect Kv channels *in vitro* [10].These accessory proteins provide an important mechanism for achieving functional diversity among potassium channels. For example, KCNE1 subunits co-assemble with KCNQ1 Kv channels to generate the I_Ks_ current in human ventricular myocardium [24], [25], and KCNE2 co assembles with hERG to form the cardiac I_Kr_ current [20]. KCNE3 has been proposed to regulate Kv3.4 α-subunits in skeletal muscle, reducing after hyperpolarizations [19]. However despite the mRNA expression of all identified KCNE genes (KCNE1-5) in mammalian brain [10], [13], [26], [27], surprisingly few studies have investigated their possible role in K^+^ channel regulation in the brain. Furthermore the possibility of the KCNE peptides regulating the sub-threshold Kv12 channels has never been addressed.

Here we firstly demonstrate that KCNE1 and KCNE3 regulate the membrane surface expression of Kv12.2 *in vitro*. Furthermore, native co-immunoprecitiation studies revealed that KCNE1 and KCNE3 interact simultaneously with Kv12.2 channels *in vivo*. This novel finding suggests that Kv12.2 channel surface expression and activity may be regulated by the formation of tripartite complexes with KCNE1 and KCNE3; thus we describe a novel role for these regulatory β subunits in the brain.

## Materials and Methods

### Ethics Statement

Mice were housed at the ICND vivarium at The Scripps Research Institute. Procedures for procurement of animals, conditioning/quarantine, housing, management, veterinary care, and disposal of carcasses were reviewed by veterinary staff and follow guidelines set down by the NIH Guide for Care and Use of Laboratory Animals.

### Molecular biology and *Xenopus* oocyte preparation

Full length mouse KCNE1 (mKCNE1), mKCNE2, mKCNE3, mKCNE4, mKCNE5 and mKv12.2 cDNAs were cloned into the pOX vector [28] for expression in *Xenopus* oocytes. Capped cRNA transcripts were prepared by run-off transcription using the T3 mMessage mMachine kit (Ambion, Austin, TX, USA). cRNAs were cleaned by lithium chloride precipitation and diluted in RNase-free dH_2_O to desired concentrations before injection. Mature *Xenopus* oocytes (Nasco, CA, USA) were isolated from ovarian lobes and defolliculated by mechanical agitation in Ca^2+^-free ND96 solution (96 mM NaCl, 2 mM KCl, 1 mM MgCl_2_, and 5 mM HEPES, with pH adjusted to 7.5 with NaOH) containing 1 mg/ml collagenase (type II, Sigma, MO, USA). Oocytes were injected with a total of 55 nl of cRNA solution in dH_2_O containing mKv12.2 (∼1 ng), and/or mKCNE cRNAs (∼50 pg) as required. For RNAi, 500 pg of double-stranded siRNA 21-mer oligos (Invitrogen, CA) were injected into oocytes immediately after injection of α-subunit Kv12.2 channel cRNA (*see* ***Table 1*** *for sequences*). Oocytes were incubated at 18°C for 48-hours before recording in ND96 solution (supplemented with 1.8 mM CaCl_2_, 100 U/ml penicillin, 100 µg/ml streptomycin, and 2.5 mM sodium pyruvate).

**Table 1:** siRNA KCNE gene sequences.

Gene	Bases	Top Strand Sequence
KCNE1	330–350	5′-GAACAAGUUUGCAGUGGAATT-3′
KCNE3	286–306	5′-GGGAACCACACGGACGCCATT-3′
KCNE5.1	260–280	5′-GGGAAUGGAAUAAGAACAATT-3′

### RT-PCR

For assessment of RNAi gene silencing at the mRNA level, equal numbers of oocytes were injected either with *Xenopus* KCNE1 siRNA (xKCNE1), xKCNE3 siRNA, or xKCNE5.1 siRNA and compared to non-injected controls (n = 6). Total RNA was extracted using an RNeasy Mini Kit (Qiagen, CA) after 48-hours incubation. RNA integrity was assessed by gel electrophoresis; only RNA preparations with two clear sharp ribosomal bands were used in subsequent experiments. First strand cDNA was synthesized from 500 ng of each total RNA sample using oligo-dT and SuperscriptIII reverse transcriptase (Invitrogen, CA). Triplicate RT reactions were performed, along with an additional reaction in which the reverse transcriptase enzyme was omitted to allow for assessment of contamination in each sample. RT-PCR for each gene was performed with primers listed in ***Table 2*** using Go Taq Hot Start polymerase (Promega, WI). The thermocycler protocol for all PCR reactions was as follows: 94°C for 3 minutes, 96°C for 20 seconds, 53°C for 30 seconds, 72°C for 45 seconds, and finally 74°C for 5 minutes; for a total of 35 cycles. To normalize for mRNA concentration, RT-PCR was also performed with *Xenopus* β-actin. Optical density of cDNA samples size-fractionated on a 1% agarose gel and stained with ethidium bromide was determined and the intensity of each cDNA product relative to β-actin was quantified using *NIH ImageJ*. RT-PCR experiments were repeated three times with separately injected oocyte batches, yielding similar results. Mean±SEM values are shown. For statistical analysis, Student's *t* test was used to determine significant difference between two groups and *p*<0.05 was considered significant.

**Table 2:** RT-PCR Xenopus (x) primer sequences.

Gene	Sense primer sequence	Antisense primer sequence
xKCNE1	5′-ATGCCAGGGTTAAACACCACTGCC-3′	5′-CTAGTTGCTGGGAGAAGAGGGGATATA-3′
xKCNE3	5′-CAGTTTGATTGGAGAGTGGGATTC-3′;	5′-TAGACCCCTGGGGCCTCGTC-3′
xKCNE5.1	5′-ATGAATTGTAGTAATACTTCTC-3′	5′-CTAAAGTAAGTTTTCACTTTCAATACT-3′
xβ-actin	5′-AAGGAGACAGTCTGTGTGCGTCCA-3′	5′-CAACATGATTTCTGCAAGAGCTCC-3′

### Kv12.2 channel antibody development and characterization

A polyclonal antibody, targeted to a highly conserved C-terminal region of the Kv12.2 channel (residues 701–802) was generated in rabbits. The corresponding DNA sequence was cloned into a pGEX-4T1 vector in frame with the GST coding sequence (Amersham, NJ). GST-Kv12.2 channel fusion proteins were produced in BL21 bacteria (Stratagene, CA) and purified via a glutathione agarose column (Amersham, NJ). The Kv12.2 channel was removed from the GST moiety by thrombin digestion, and conjugated to KLH. Kv12.2-KLH conjugated antigen was used for the initial boost, and unconjugated Kv12.2 antigen was provided for three subsequent boosts. All immunizations and bleeds were performed by QED Biosciences (CA). Antibody specificity was rigorously tested and optimized in Kv12.2 channel transiently-transfected and non-transfected cell lines. Briefly, CHO cells were plated at a density of ∼5×10^6^ cells per 100 mm Petri dish, cells were washed once in ice cold PBS and ∼2 ml of homogenization buffer (10 mM Tris-HCl, 320 mM sucrose, and a protease inhibitor cocktail (Calbiochem, San Diego, CA). was added to each Petri dish. Cells were removed with a cell scraper and homogenized with an 18-guage needle, passing the cell lysate through the needle 10 times on ice. Lysates were centrifuged (1000×*g*, 10 min), supernatants were collected on ice and quantified by SDS-PAGE and Coomassie blue protein gel stain (Pierce, Rockford, IL). Equal concentrations of protein were loaded onto a 12% denaturing SDS Tris-glycine gel (Invitrogen) and following transfer blots were probed with Kv12.2 primary antibody using standard Western blot techniques. Antibody validation studies with controls revealed that 1∶100 dilution of the Kv12.2 primary antibody gave the best signal to noise ratio.

### Membrane surface biotinylation assays

Kv12.2 channel membrane expression was assessed by membrane surface biotinylation assay utilizing a ‘Cell surface protein isolation kit’ (Pierce, IL), according to instructions from the manufacturer. *Xenopus* oocytes (10 per condition) were injected with Kv12.2 channel RNA alone, or Kv12.2 RNA plus KCNE1 siRNA and/or KCNE3 siRNA. To assess levels of endogenous channel expression, oocytes were injected with dH_2_0 only. 48 hours post-injection oocytes were mechanically stripped of their vitelline membranes and surface proteins were labeled with sulfo-NHS-Biotin (Pierce, IL) for 30 minutes at 4°C; the reaction was then quenched and the oocytes were gently washed with Tris-buffered saline (TBS). Oocytes were lysed in 500 µl of radio-immunoprecipitation assay (RIPA) buffer (1 mM EDTA, 20 mM Tris, 158 mM NaCl, 0.5% NP-40, 0.5% sodium deoxycholate) supplemented with protease inhibitors, and homogenized using a 5 ml syringe with a 22-guage needle. Homogenized lysates were then left on ice for 30 minutes and, to improve the solubilization efficiency, were passed through a 5 ml syringe with a 27-guage needle five further times. Lysates were centrifuged (10,000×*g*) for 2 minutes at 4°C, the clarified supernatant was collected and 200 µl of each supernatant was saved to assess changes in oocyte total protein expression. Biotin labeled proteins were isolated by immobilization on a *NeutrAvidin Gel* column (Pierce, IL), and eluted with 2 mM free D-biotin (Pierce, IL); isolated samples were then analyzed by SDS-PAGE, transferred to nitrocellulose membranes, and probed with primary antibodies as indicated for one hour at room temperature. Detection was via chicken anti-rabbit or chicken anti-mouse horseradish peroxidase-coupled (HRP) secondary antibodies (Bio-Rad, CA) with one-hour incubation at room temperature, and membranes were developed with ‘Supersignal ECL’ (Pierce, IL). To assess that only the membrane fraction of the oocytes had been successfully isolated the endoplasmic reticulum (ER) marker calnexin (Stressgen, MI; 1∶2000) was used to re-probe each blot. Finally, the cell membrane marker integrin-β1 (1∶2000) (N-20, Santa Cruz Biotechnology, CA) was used to ensure equal protein loading. The intensity of each band relative to the total amount of protein loaded per lane (β-actin) or total membrane fraction (β1-intergrin) was quantified using the ‘gel’ module in *NIH ImageJ*. Mean±SEM values are shown. For statistical analysis, Student's *t* test was used to determine significant difference between two groups and *p*<0.05 was considered significant.

### Co-immunoprecipitation of Kv12.2 channels from mouse brain lysates

Freshly isolated adult mouse brains were homogenized using a ‘Polytron’ homogenizer (power 18) for 2×10−15 s pulses on ice. A gap of 1 minute was allowed between each pulse, to ensure the sample remained cool. The homogenate buffer was composed of 10 mM Tris-HCl (pH 7.4), containing 320 mM sucrose plus a protease inhibitor cocktail (Calbiochem, CA). Homogenates were centrifuged at 1000×*g* for 10 minutes at 4°C; the supernatant was removed and centrifuged further (100,000 *g*) for 30-minutes at 4°C. The pellet was solubilized in immunoprecipitation (IP) buffer (50 mM Tris-Cl, 20 mM MgCl_2_, 150 mM NaCl, 0.5% Igepal (Sigma, CA) and a protease inhibitor cocktail (Sigma), pH 7) at 4°C for 30 minutes (for details see Manning 1999). Anti-Kv12.2 (1∶100), KCNE1 (N-16) or KCNE3 (N-18) (both Santa Cruz Biotechnology, CA; 1∶100) or anti-Kir2.1 (Chemicon CA; 1∶50) as a negative control, covalently conjugated to 20 µl of protein A/G sepharose beads (ProFound kit, Pierce, IL) were added to each respective preparation, and left overnight at 4°C. Each lysate/antibody/bead preparation was then transferred to a preconditioned spin column and centrifuged at 16,000×g for 1 minute. Each column was rinsed four times with 500 µl IP buffer; proteins were eluted with 30 µl ‘sample buffer’ (Cytosignal) and incubated at room temperature for 15 minutes before centrifugation at 20,000×*g* for 2 minutes. Samples were denatured with β-mercaptoethanol (710 mM) and heat (95°C for 1 minute) before loading onto a 12% SDS polyacrylamide gel (Invitrogen, CA). Proteins were transferred to nitrocellulose membranes (Amersham Pharmacia, PA), blocked with ‘SuperBlock’ buffer (Pierce, IL) for 1 hour, and then probed with Kv12.2 (1∶200) antibody for 1 hour at room temperature. Secondary antibody chicken-anti-rabbit-HRP (Santa Cruz, CA; 1∶5000) was applied for 1 hour at room temperature, and the membrane was developed with Supersignal ECL (Pierce, IL). The membrane was then repeatedly stripped with Western blot stripping buffer (Pierce, IL) and probed sequentially with anti-KCNE1 (1∶200), anti-KCNE3 (1∶200), and finally anti-Kir2.1 (1∶200) as a negative control. Co-immunoprecipitation experiments were performed three times yielding similar results.

For native co-immunoprecipitation experiments whole mouse brains were homogenized according to the ‘NativePAGE sample preparation kit’ (Invitrogen) instructions. Briefly, mouse whole brain (∼10 mg) was homogenized in 1 ml of NativePAGE sample buffer (plus 1% digitonin and a protease inhibitor cocktail) by sonication on ice (3×15 seconds each at ∼50% power). The lysate was centrifuged (20,000 g) for 30-minutes at 4°C, and once clarified lysate protein concentration was determined by BCA (Invitrogen) assay. Co-immunoprecipitation experiments were conducted as above with the following modifications, lysate (20 µl) was added directly to each respective antibody bound (KCNE1/KCNE3 or IRK1) protein A/G sepharose column and left overnight at 4°C. The column was subsequently washed and proteins were eluted with the NativePAGE sample buffer, 50% of the eluted protein was directly applied to a ‘second-round co-immunopreciptiation assay’ with the channel Kv12.2 antibody bound to the column, and the remaining 50% reserved for direct gel electrophoresis analysis. G-250 (0.25%) sample additive (Invitrogen) was added to each protein preparation prior to loading onto a 4-16% ‘NativePAGE Novex Bis-Tris Gel’ (Invitrogen). Electrophoresis with blue ‘NativePAGE Cathode Buffer’ (Invitrogen) and ‘NativePAGE Anode Buffer’ both at pH 7 (Invitrogen) was begun at 10 mA at 4°C for 1.5 hours. After 1.5 hours, the cathode buffer was replaced by anode buffer, and electrophoresis continued for a further 5 hours at 8 mA (120 V). Proteins were transferred overnight at 4°C in ‘NuPAGE Transfer Buffer’ (Invitrogen) to a PVDF membrane (Amersham Pharmacia, PA), blocked with ‘SuperBlock’ buffer (Pierce, IL) for 1 hour, and probed with respective antibodies as above.

### Electrophysiology

Macroscopic currents were recorded from oocytes using standard two-electrode voltage-clamp (CA-1B amplifier (Dagan Corp., MN)), sampled at 2 KHz and filtered at 1 KHz. Data were collected using the pCLAMP/Digidata acquisition package (Axon Instruments, CA), and subsequently analyzed using Clampfit (Axon instruments, CA) and Origin (OriginLab, MA). Micro-electrodes contained 3 M KCl and had tip resistances between 0.5–1 MΩ. Oocytes were continually perfused with extracellular solution at room temperature containing: 98 mM Na-methanesulfonate (MES), 2 mM K-MES, 2 mM CaCl_2_, 10 mM HEPES, pH 7.0. The low [Cl^−^] of the recording solutions was sufficient to eliminate contaminating outward native Cl^−^ currents. Agar bridges (1 M NaCl) were used to isolate bath clamp circuitry. Peak currents were measured at each voltage, and conductance/voltage (GV) parameters are averages derived from Boltzmann fits of isochronal tail currents recorded at −60 mV (G = G_max_/[1+exp(V - V_0.5_/*k*)], where V_0.5_ is the half-maximal activation, and *k* is the slope factor). All values are mean±SEM (n≥6), statistical significance was determined by Students *t* test for unpaired observations; significance was taken as *P*<0.05.

## Results

### Kv12.2 channel currents in *Xenopus* oocytes are suppressed by endogenous KCNE1 and KCNE3

**Figure 1:**
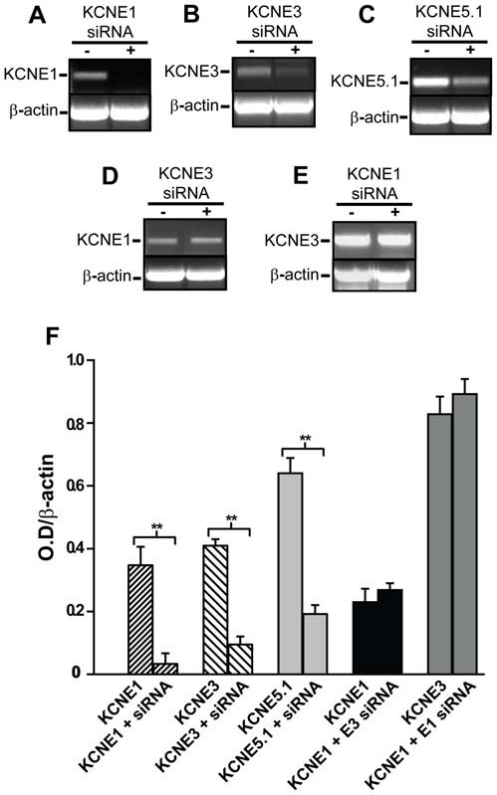
Knockdown of endogenous KCNE expression in *Xenopus* oocytes with siRNA. (A–E) RT-PCR of xKCNE1, xKCNE3 and xKCNE5.1 mRNA isolated from *Xenopus* oocytes injected with dH_2_O (−) or the indicated siRNA (+). RT-PCR for β-actin is shown as a control for normalization of band intensity. (A–C) siRNAs designed against xKCNE1, xKCNE3 and xKCNE5.1 all significantly knock down mRNA expression level of their respective target genes. (D–E) In contrast, siRNA targeted to xKCNE1 and xKCNE3 do not affect mRNA expression levels of other KCNE genes. (F) Optical density of cDNA bands relative to β-actin was quantified using *NIH ImageJ*. xKCNE1, xKCNE3 and xKCNE5.1 siRNA bands indicted a ∼10-fold, ∼4-fold, and ∼4fold reduction in expression of each respective gene; control injections did not show a significant reduction. Each experiment was performed 3 times; values show Mean±SEM, ** *p*<0.01.

Previous studies have demonstrated that KCNE1, KCNE3, and KCNE5.1 are endogenously expressed in *Xenopus* oocytes [29]. We therefore investigated whether these endogenous K^+^ channel β-subunit regulatory proteins modulate heterologously expressed Kv12.2 channels. RNA interference (siRNA) was firstly utilized to specifically knockdown endogenous KCNE1, KCNE3 or KCNE5.1 expression. Double-stranded xKCNE-specific siRNA oligos designed by Anantharam *et al*. (2003) [29] were injected into *Xenopus* oocytes to assess their ability to knock down xKCNE mRNA expression. The band intensity of amplified KCNE1 cDNA from oocytes injected with xKCNE1 siRNA was almost non-detectable (Figure 1A, F). Similarly, xKCNE3 and xKCNE5.1 siRNA significantly reduced KCNE3 and KCNE5.1 cDNA expression. (Figure 1B, C, F). To ensure the quality and equal cDNA concentrations between samples, results were normalized with RT-PCR for *Xenopus* β-actin cDNA. We also examined whether xKCNE1 siRNA affected xKCNE3 RNA levels and *vice versa*. No detectable gene knockdown of either KCNE1 or KCNE3 was observed by these experiments, demonstrating the specificity of each respective siRNA (Figure 1D–F).

**Figure 2:**
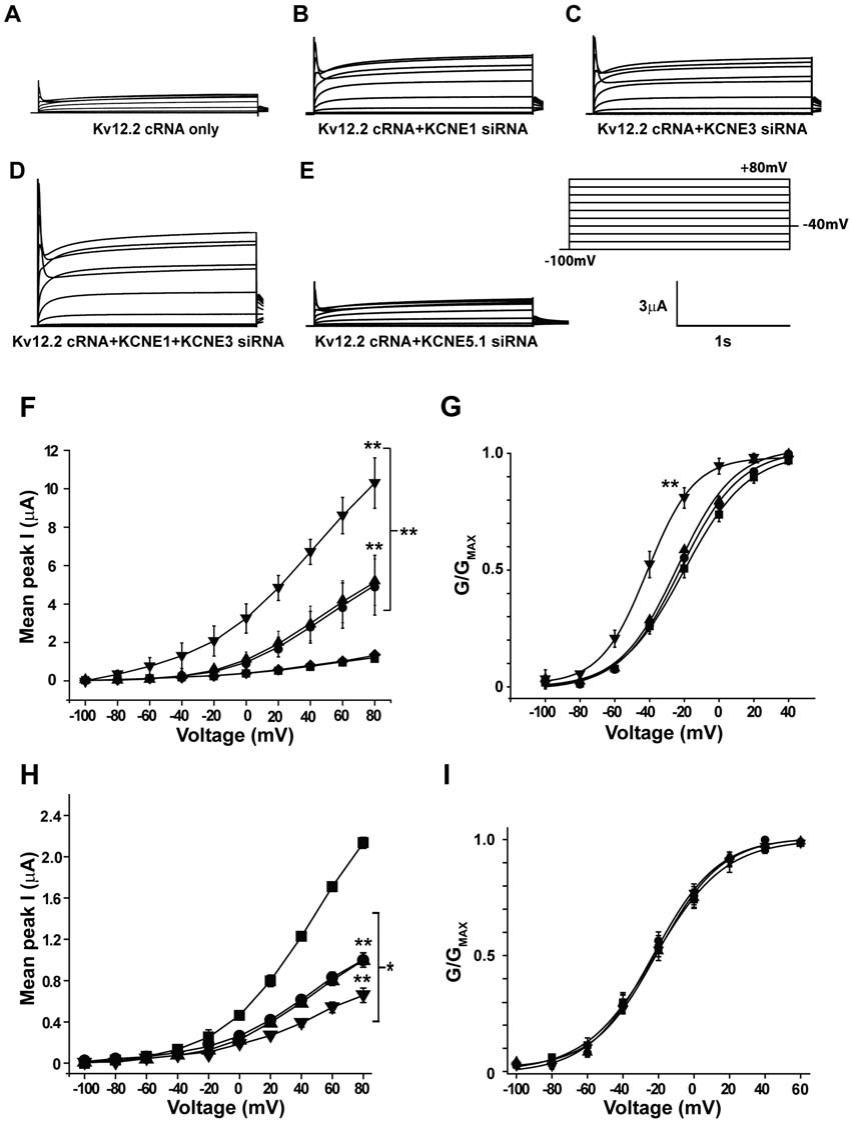
KCNE1 and KCNE3 inhibit Kv12.2 currents in *Xenopus* oocytes. (A–E) Current traces recorded from oocytes injected with (A) Kv12.2 cRNA only, or co-injected (B) KCNE1, (C) KCNE3, (D) KCNE1 and KCNE3, or (E) KCNE5.1 siRNAs. Currents were recorded in response to 2 s voltage steps from −100 to +80 mV, in 20 mV increments from a holding potential of −100 mV; tail currents were recorded at −40 mV (protocol in *inset*). (F) Peak current-voltage relationships from oocytes injected with Kv12.2 cRNA only (▪), or Kv12.2 cRNA plus either KCNE1 (•), KCNE3 (▴), KCNE1 and KCNE3 (▾), or KCNE5.1 (♦) siRNAs. KCNE1 and KCNE3 siRNAs significantly increase Kv12.2 currents and have an additive effect in combination. (Mean±SEM, n = 12–16, ** *p*<0.01). (G) Normalized conductance voltage (GV) curves measured from isochronal tail currents for oocytes injected with Kv12.2 cRNA only (▪), and either KCNE1 (•), KCNE3 (▴), or KCNE1+KCNE3 (▾) siRNA. Lines show Boltzmann fits; parameters are given in the *Results*. Only dual injection of xKCNE1 and xKCNE3 siRNAs caused a significant shift in V_50_. Data are given as Mean±SEM, n = 12–16, (** *p*<0.01). (H) Peak current-voltage relationships from oocytes injected with Kv12.2 cRNA (▪), or Kv12.2 cRNA+mKCNE1 (•), mKCNE3 (▴), mKCNE1 and mKCNE3 (▾) cRNA (protocol as in A, Mean±SEM, n = 8, * *p*<0.05, ** *p*<0.01). Co-injection of Kv12.2 with mKCNE1 and/or mKCNE3 cRNA significantly reduced Kv12.2 currents. (I) GV curves from isochronal tail currents recorded from oocytes injected with Kv12.2 cRNA (▪), or Kv12.2 and either mKCNE1 (•), mKCNE3 (▴), and mKCNE1 and mKCNE3 (▾) cRNA. Boltzmann fits (lines) revealed that the voltage-dependence of Kv12.2 activation was not significantly affected by overexpression of KCNE1 and KCNE3. V_50_ values are given in the *Results* (n = 8).

We next determined the effect of attenuating endogenous KCNE1, KCNE3, and KCNE5.1 on Kv12.2 channel currents. Two electrode voltage clamp recordings were performed 48-hours post injection of Kv12.2 channels, in the absence or presence of the respective KCNE siRNA. Kv12.2 channel heterologous expression resulted in a relatively small voltage-dependent outward current (1.2±0.11 µA peak current at 80 mV; V_50_ -21.9±0.81 mV, Figure 2A, F–G). Typical of Kv12.2 channel currents, when the voltage was stepped to potentials above 20 mV the outward current develops a visible rapidly inactivating component [2], [3]. KCNE1 and KCNE3 siRNAs significantly increased Kv12.2 currents by ∼4.1 fold and ∼4.2-fold, respectively (Figure 2B–C, F; p<0.01). Furthermore the combination of KCNE1 and KCNE3 siRNAs increased Kv12.2 channel currents ∼9-fold (Figure 2D, F, p<0.01), suggesting an additive effect of each KCNE β-subunit. In contrast, KCNE5.1 siRNA knockdown had no significant effect on Kv12.2 currents, compared to Kv12.2 cRNA injected only controls (Figure 2E–F). Analysis of isochronal tail currents revealed that the voltage for Kv12.2 channel activation (GV) was not significantly affected by either xKCNE1 (V_50_ −22±1.14 mV) or xKCNE3 (V_50_ −24±1.32 mV) siRNAs when compared to dH_2_O injected controls (V_50_ −21.9±0.81 mV, Figure 2G). However, the GV was significantly left shifted by ∼20 mV by dual injection of xKCNE1 and xKCNE3 siRNAs (V_50_ −41.2±0.8 mV, Figure 2G). Previous studies have shown that KCNE peptides modulate K^+^ channel activation and/or deactivation kinetics; for example, KCNE1 slows activation and deactivation of KCNQ1 [24], [25], and KCNE3 slows activation and deactivation of Kv2.1 channels in neurons [30]. Therefore, we repeated the experiments on a non-inactivating mutant Kv12.2 channel (S464T), which contained a single point mutation in the P region of the channel [14], to better assess the effect of dual knockdown of KCNE1 and KCNE3 on Kv12.2 channel kinetics. We did not find significant changes in either activation or deactivation, but did observe significant increases in current magnitude and a similar shift in GV as for the wild-type channel with the dual injection of xKCNE1 and xKCNE3 siRNAs, indicating that the S464T mutation did not disrupt the channel interaction with KCNE1 and KCNE3 (data not shown). These findings suggest that KCNE1/KCNE3 β subunits primarily regulate Kv12.2 channels via control of current amplitude and activation threshold.

### Kv12.2 currents are reduced by over-expression of KCNE1 and KCNE3 in *Xenopus* oocytes

Consistent with xKCNE siRNA gene knockdown studies, co-injection of Kv12.2 with mKCNE1 or mKCNE3 cRNA significantly reduced Kv12.2 currents>2-fold (from 2.23±0.02 µA, V_50_ −22.8±0.12 mV to 0.9±0.07 µA, V_50_ −22.5±2.17 mV, or 0.9±0.06 µA, V_50_ −20.2±2.1 mV, respectively) (Figure 2H–I). Similarly, the co-injection of both mKCNE1 and mKCNE3 cRNA further inhibited Kv12.2 currents (∼3-fold; 0.73765±0.1375 µA, V_50_ −21.6±0.9 mV) (Figure 2H–I). Consistent with siRNA studies, co-expression of mKCNE5 cRNA did not affect Kv12.2 currents (data not shown). Injection of KCNE1, KCNE3, or KCNE5 cRNA alone into oocytes did not generate currents significantly different to H_2_O-injected controls in our protocols (data not shown). Previous studies have demonstrated that injection of KCNE1 β-subunits into oocytes generates a small, slowly activating *I*_Ks_-like current via assembly with endogenous KCNQ1 [24], [25]. We probably did not detect a significant amount of this current in our experiments because we used much shorter voltage steps and shorter incubation periods. Taken together these data suggest that KCNE1 and KCNE3 but not KCNE5 β-subunits, significantly reduce Kv12.2 channel currents via a mechanism that has only modest effects on channel gating. Furthermore these studies suggest that the ability of KCNE1 and KCNE3 to modulate Kv12.2 channels is conserved across vertebrate species.

### Kv12.2 channel antibody generation

**Figure 3:**
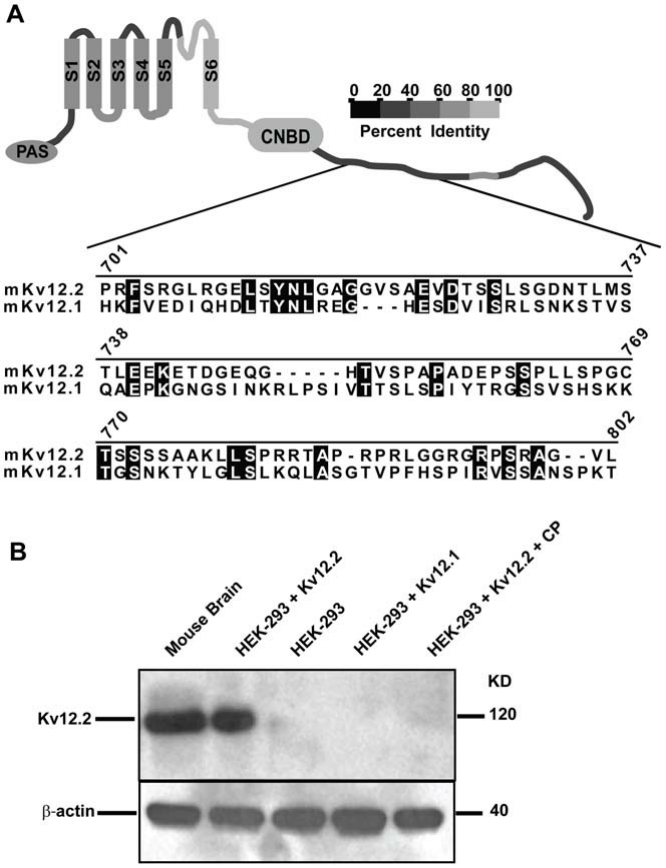
Characterization of the Kv12.2 channel antibody. (A) Alignment of a 100 amino acid section, 702–801 of the cytoplasmic C-terminus of mouse Kv12.2 used to generate a Kv12.2-specific polyclonal antibody in rabbit to Kv12.1. The homologous region of Kv12.3 (not shown), is most similar to Kv12.1. Amino acid identities are shaded and numbers indicate amino acid position in Kv12.2. The cartoon depicts a Kv12.2 subunit with 6 transmembrane domains (S1–S6), a Per-Arnt-Sim (PAS) motif and a putative cyclic nucleotide binding motif (cNBD). Gray scale coding indicates the level of amino acid identity shared between Kv12.2 and other Kv12 channels in each region of the channel. (B) *Top panel*, Western blot analysis demonstrates that the Kv12.2 antibody (anti-Kv12.2) recognizes a single band of ∼120 KD (the predicted size of a Kv12.2 channel monomer) from mouse brain and HEK-293 cells transiently transfected with mKv12.2 channel cDNA (HEK-293+Kv12.2). Anti-Kv12.2 did not recognize specific bands in non-transfected HEK-293 cell lysates (HEK-293), in HEK-293 cells transfected with the closely related Kv12.1 channel (HEK-293+Kv12.1), or when excess antigenic control peptide (CP) was present to block immunodetection (HEK-293+Kv12.2+CP). *Bottom panel*, anti-β-actin demonstrates equal protein loading for each condition. These experiments were repeated 4 times yielding similar results.

A 100 amino acid section (position 702–801) of the cytoplasmic C-terminus of mouse Kv12.2 was used to generate a Kv12.2-specific polyclonal antibody in rabbit. The region was selected for high predicted antigenicity and lack of conservation with other Kv12 channels (Figure 3A). Western blot analysis demonstrated that the Kv12.2 antibody recognized a single band of ∼120 KDa (the predicted size of a Kv12.2 monomer) on a denaturing gel in lysates from mouse brain and HEK-293 cells transiently transfected with Kv12.2 (Figure 3B). The Kv12.2 antibody (anti-Kv12.2) did not recognize specific bands in non-transfected HEK-293 cell lysates, or in HEK-293 cells transfected with the closely related Kv12.1 channel, or when excess antigenic peptide was present to block immunodetection (Figure 3B). The antigenic peptide also blocked detection of Kv12.2 in mouse brain lysates in separate experiments (data not shown). The antibody therefore appeared to be highly specific for Kv12.2 channels as no immunoreactivity was evident in the above conditions.

### Biotinylation assays reveal that KCNE1 and KCNE3 gene knock-down increases Kv12.2 channel membrane surface expression

**Figure 4:**
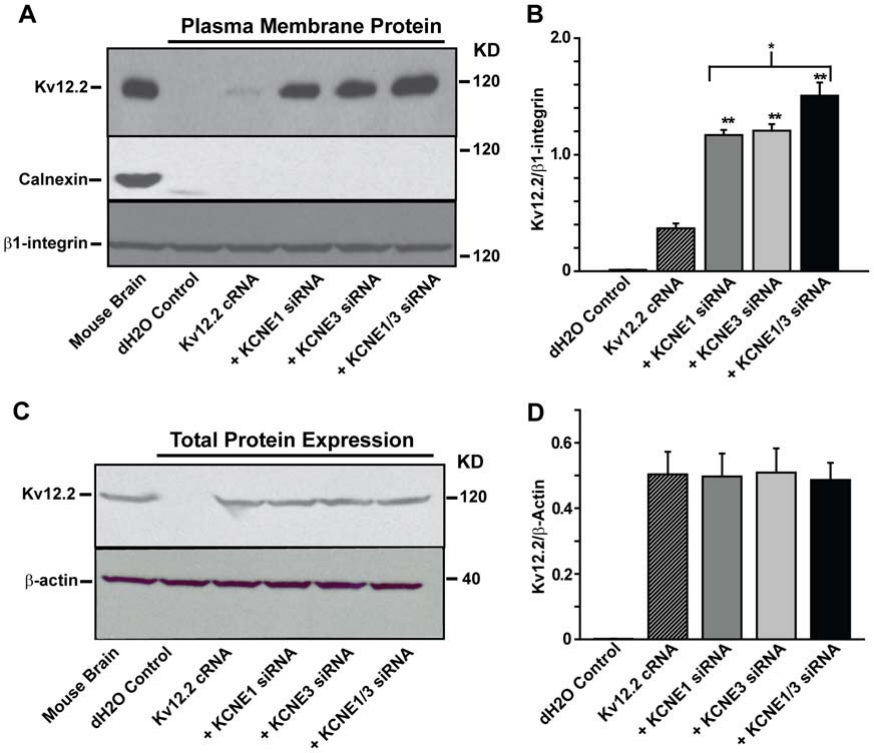
KCNE1 and KCNE3 reduce membrane surface expression of Kv12.2 channels. (A) Blots of the biotinylated plasma membrane fraction of proteins from *Xenopus* oocytes injected with Kv12.2 cRNA alone or in combination with KCNE1 and/or KCNE3 siRNA subjected to membrane surface biotinylation. *Top panel*, Detection of Kv12.2 channel protein with anti-Kv12.2. *Xenopus* oocytes injected with Kv12.2 cRNA showed just detectable membrane surface expression; whereas the co-injection of KCNE1 and/or KCNE3 siRNA dramatically increased Kv12.2 channel plasma membrane expression. Kv12.2 was not detected in oocytes injected with dH_2_O (negative control), and was robustly detected in mouse whole brain lysate (positive control). An endoplasmic reticulum (ER) marker (calnexin, *middle panel*), and a cell membrane marker (β1-integrin, *bottom panel*), were used as a negative and positive controls, respectively, to show specific isolation of the plasma membrane protein fraction and to confirm equal protein loading. (B) The optical density of each Kv12.2 protein band was quantified using *NIH ImageJ* and normalized to density of β1-integrin for comparison; both xKCNE1 or xKCNE3 siRNA increased Kv12.2 channel membrane expression>3-fold (Mean±SEM, n = 4, ** *p*<0.01). Furthermore, the combination KCNE1 and KCNE3 siRNA increased surface Kv12.2 expression ∼20% further than either siRNA alone (n = 4, * *p*<0.05). (C) Blots showing total protein expression from *Xenopus* oocytes injected as in A. *Top panel*, Kv12.2 channel total expression was not detected in dH_2_O-injected oocytes, and did not increase when Kv12.2 was injected with siRNA for KCNE1 and/or KCNE3. *Bottom panel*, β-actin was used to confirm equal protein loading for each condition assessed. (D) The optical density of each Kv12.2 protein band was quantified using *NIH ImageJ* and normalized to β-actin; xKCNE1, xKCNE3 or KCNE1 and KCNE3 siRNA did not significantly affect total Kv12.2 channel expression (Mean±SEM, n = 4).

We next investigated the mechanism by which KCNE1 and KCNE3 attenuated Kv12.2 currents. KCNE subunits have been proposed to directly affect channel conductance, but recent studies in HEK-293 cells interestingly revealed that KCNE1 and KCNE2 regulate HERG channel surface expression on the plasma membrane [31]. We therefore hypothesized that KCNE1 and KCNE3 are important for Kv12.2 channel trafficking. To investigate this possibility we biotinylated membrane surface proteins in *Xenopus* oocytes injected with Kv12.2 channel cRNA alone and in combination with KCNE1 and/or KCNE3 siRNA. Retrieval of the tagged proteins allowed us to compare total and membrane surface expression of Kv12.2 protein for each case. Oocytes injected with Kv12.2 cRNA showed just detectable cell surface expression; whereas the co-injection of KCNE1 and/or KCNE3 siRNA dramatically increased the amount of biotinylated Kv12.2 channel plasma membrane expression (all ∼3-fold increase, p<0.01) (Figure 4A, B). The magnitude of this increase is consistent with our previous observations of Kv12.2 current increases observed with KCNE1 or KCNE3 siRNA knockdown, suggesting that this change in surface expression accounts for the changes in current amplitude. Furthermore, we observed an additional significant increase in Kv12.2 channel plasma membrane expression with the co-injection of both KCNE1 and KCNE3 siRNA (∼22.7% P<0.05 for KCNE1 alone, and ∼20% P<0.05, for KCNE3 alone, Figure 4A, B). We speculate that this further increase in channel protein expression is less than expected based on electrophysiology because of saturation of detection sensitivity in the ECL-based immunoassay. In comparison, electrophysiological measurements are linear over a wide range and therefore likely provide a better quantitative measure for changes in Kv12.2 surface expression. Kv12.2 channel plasma membrane expression was not detected in water-injected oocytes (Figure 4A, B), and importantly the biotinylated membrane fraction did not appear contaminated with endoplasmic reticulum (ER) proteins, as the specific ER marker calnexin was not detected (Figure 4A *middle panel*). The enrichment of Kv12.2 channel protein in the biotinylated plasma membrane fraction was confirmed using β1-integrin as a plasma membrane marker (Figure 4A, *bottom panel*).

In addition to determining the effect of KCNE peptides on Kv12.2 channel plasma membrane surface expression, we assessed changes in total channel protein expression. Determining the effects of the KCNE β-subunits on total channel protein were important as previous studies have shown that KCNE4 and KCNE5 decreased KCNQ plasma membrane expression by reducing total channel protein expression [32]. Importantly no changes were observed in the total amount of the channel protein following KCNE1 and/or KCNE3 co-injection, when compared to Kv12.2 channel injected alone (Figure 4C, D). The increase in Kv12.2 channel expression is therefore specific to the plasma membrane, and not due to a non-specific increase in total oocyte Kv12.2 channel protein expression.

In summary we demonstrate that KCNE1 or KCNE3 gene knockdown significantly increased Kv12.2 channel membrane expression, and moreover the dual knockdown of both regulatory β-subunits significantly increased further the channel membrane fraction. Taken together these studies suggest that endogenous KCNE1 and KCNE3 are important for the regulation of Kv12.2 trafficking to the plasma membrane and subsequent channel activation.

### KCNE1 and KCNE3 interact with Kv12.2 channels *in vivo*

**Figure 5:**
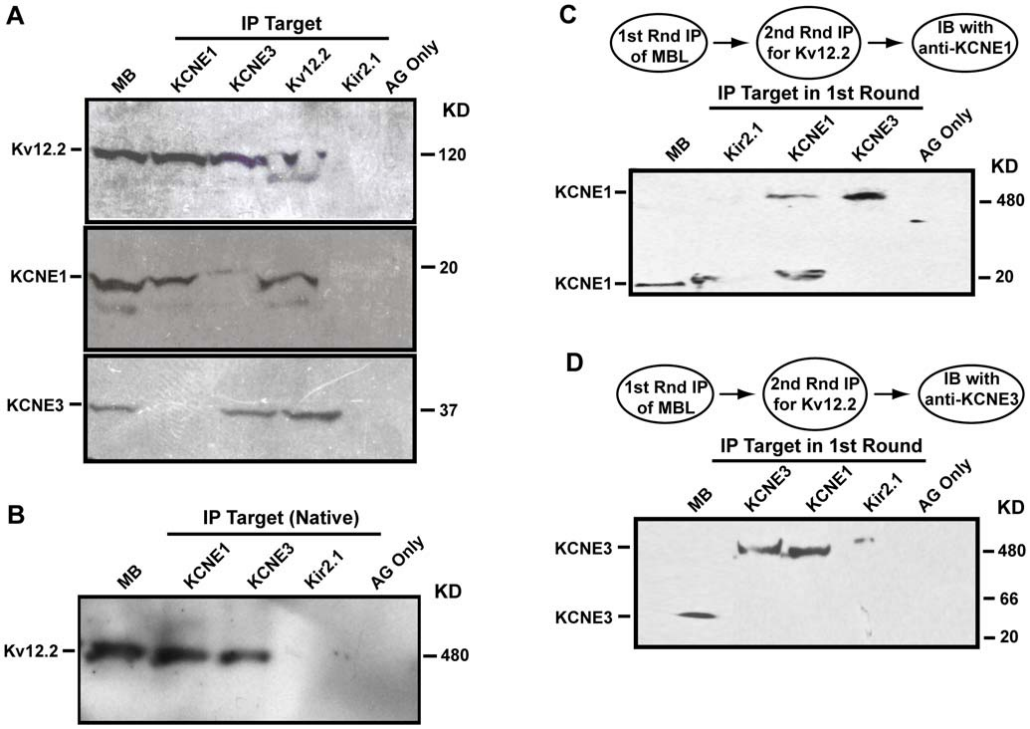
Kv12.2 channels simultaneously associate with KCNE1 and KCNE3 β-subunits *in vivo*. (A) Mouse brain lysates (MB) were immunoprecipitated (IP) with anti-KCNE1, anti-KCNE3, anti-Kv12.2, or anti-Kir2.1 and were then subjected to SDS-PAGE and Western Blot analysis. Whole mouse brain lysate (MB) and proteins precipitated with unconjugated beads (AG only) are shown as positive and negative controls, respectively. Proteins used for IP are indicated at the top, proteins detected in Western blot (IB) are indicated at the left. *Top panel*, IB with anti-Kv12.2 indicates that both KCNE1 and KCNE3 interact *in vivo* with Kv12.2 channels. *Middle panel*, IB was stripped and re-probed with anti-KCNE1. KCNE1 immunoprecipitates with Kv12.2 but not KCNE3. *Bottom panel*, IB was then re-stripped and re-probed with anti-KCNE3. KCNE3 immunoprecipitates with Kv12.2 but does not interact with KCNE1. Controls show little or no IP of Kv12.2, KCNE1 or KCNE3 with anti-Kir2.1 or unconjugated beads. (B–D) Two-step co-immunoprecipitation assays run under native protein conditions. The Kv12.2 channel complex labeled under native conditions was ∼500 Kd, consistent with a channel tetramer. (B) Kv12.2 channels were immunoprecipitated first with anti-KCNE1 or anti-KCNE3 in these native protein conditions. Specificity was assessed using the anti-Kir2.1 antibody and unconjugated beads as negative controls. Proteins immunoprecipitated in this first IP were subjected to a second IP using anti-Kv12.2. (C) Anti-KCNE1 detected the Kv12.2 tetramer complex after the second IP regardless of whether anti-KCNE1 or anti-KCNE3 was used for the first IP. (D) Similarly, anti-KCNE3 detected the Kv12.2 complex after the second IP with anti-Kv12.2 even if anti-KCNE1 was used for the first IP. These results can be explained if KCNE1 and KCNE3 simultaneously interact with individual Kv12.2 channels. Denatured mouse brain lysate was loaded into the first lane, to confirm the established size of the respective KCNE β-subunit. Note that some KCNE1 has dissociated from the channel complex in the KCNE1 lane of (C).

Next we questioned whether endogenous KCNE1 and/or KCNE3 β-subunits interact with neuronal Kv12.2 channels in the brain by looking for Kv12.2/KCNE protein complexes *in vivo*. Mouse brain lysates were immunoprecipitated with the following antibodies: anti-KCNE1, anti-KCNE3, or anti-Kv12.2, and the immunoblots were probed with anti-Kv12.2. Immunoblot indicated that both KCNE1 and KCNE3 interact directly *in vivo* with Kv12.2 channels (Figure 5A, *top panel*). The specificity of this interaction was demonstrated using anti-Kir2.1 antibody as a negative control; Kir2.1 is an inwardly rectifying potassium channel that is highly expressed in the brain [33], and does not interact with KCNE1 [34]. In our experiments, Kir2.1 immuno-reactivity was evident in mouse brain (data not shown); however it did not demonstrate significant binding to KCNE1, KCNE3 or Kv12.2 channels (Figure 5A). To further confirm the co-immunoprecipitation results we performed reciprocal experiments using anti-Kv12.2 as the ‘pull-down’ antibody; the blots were probed with either anti-KCNE1 or anti-KCNE3. Consistent with the KCNE1 and KCNE3 co-immunoprecipitation data, anti-Kv12.2 ‘pull-down’ experiments revealed that both KCNE1 and KCNE3 immunoprecipitate with Kv12.2 channels (Figure 5A *middle and bottom panels*, respectively).

To further characterize the KCNE1/KCNE3 β-subunit interaction with Kv12.2 channels we performed a two-step co-immunoprecipitation assay under native protein conditions. The main aim for conducting these additional co-immunoprecipitation experiments was to determine whether KCNE1 and KCNE3 β-subunits simultaneously interact with individual tetrameric Kv12.2 channels in neurons. Consistent with previous data, under native conditions the tetrameric Kv12.2 channel immunoprecipitates with KCNE1 or KCNE3 β-subunits; and again the specificity of this interaction was assessed using the anti-Kir2.1 antibody as a negative control (Figure 5B). The size of the Kv12.2 channel complex was ∼500 Kd, which is consistent with a tetrameric pore structure and allows for small associated proteins such as KCNE1 and KCNE3. The migration of KCNE1 and KCNE3 with this large Kv12.2 channel complex is strong additional proof for specific interaction of these proteins *in vivo* and importantly shows that channel structure is maintained under the conditions we used for co-immunoprecipitation assays. This allowed us to ask whether KCNE1 and KCNE3 simultaneously interact with individual tetrameric Kv12.2 channels, since it is believed that multiple, and most likely two, KCNE subunits associate with each tetrameric potassium channel [35], [36]. Since KCNE1 and KCNE3 do not interact directly, such a scenario could only be detected if tetrameric channel structure is maintained during immunoprecipitation assays. The relatively small size of the KCNE1 and KCNE3 subunits, 14 kDa and 36 kDa respectively, makes it impossible to accurately determine the stoichiometry of KCNE/Kv12.2 interactions based on the Kv12.2 channel complex size. Therefore, to determine whether both KCNE1 and KCNE3 β-subunits can interact with tetrameric Kv12.2 channels simultaneously, we conducted a ‘second- round’ co-immunoprecipitation experiment, whereby the proteins from the ‘first-round’ KCNE1 or KCNE3 ‘pull-down’ were subsequently subjected to pull-down by anti-Kv12.2. Blots were then probed with either anti-KCNE1 or anti-KCNE3. Following the ‘second-round’ co-immunoprecipitation with anti-Kv12.2, KCNE3 protein could be detected in samples where anti-KCNE1 was used for the ‘first-round’ pull-down. In addition the reciprocal interaction occurred when anti-KCNE3 was utilized in the ‘first-round’ co-immunoprecipitation and the final blot was probed for presence of KCNE1 (Figure 5C, D). These two-step co-immunoprecipitation experiments were repeated 3 times with similar results. The results suggest that KCNE1 and KCNE3 β subunits form tripartite channel complexes with Kv12.2 *in vivo*.

## Discussion

We demonstrate here that the K^+^ channel β-subunits KCNE1 and KCNE3 regulate trafficking and activation of Kv12.2. This is the first evidence that KCNE subunits associate with Kv12 family potassium channels. Furthermore, we show by native co-immunoprecipitation experiments that both β-subunits, KCNE1 and KCNE3, simultaneously interact with tetrameric Kv12.2 channels *in vivo*; suggesting the existence of a native channel tripartite complex (Kv12.2-KCNE1-KCNE3). As Kv12.2 channels are highly expressed in several important brain regions, including the hippocampus, amygdala and cerebral cortex [5], [8], we predict that *in vivo* association of KCNE1 and KCNE3 with Kv12.2 channels has considerable significance to the modulation of neuronal excitability. The specific localization of KCNEs in the brain has not been carefully defined; however northern blot analysis in human brain has demonstrated that KCNE3 is expressed in the hippocampus, cerebellum, thalamus, hypothalamus and cerebral cortex [30] and has therefore has specific overlap with areas high in Kv12.2 channel expression [8]. It is now well established that KCNQ1 channels assemble with KCNE β-subunits for correct physiological function; mutations that disrupt this complex formation result in congenital deafness and inherited cardiac arrhythmias [10], [37], [38]. Interestingly studies in the heart have shown that the expression of the KCNE β-subunits fluctuates during disease states, suggesting that a balance in the variety of KCNE β-subunits may be required to physiologically regulate K^+^ channels [39]. Our data suggest that a similar regulatory mechanism may exist in the brain.

Possible roles for KCNE β subunits in the regulation of other neuronal K^+^ channels have been proposed. Furthermore the mRNA transcripts for all five KCNE β-subunits have been detected in tissue from rodent brain [10]. McCrossan *et al*. (2003) [14] have demonstrated that Kv2.1 and Kv3.1 immunoprecipitate from rat brain membranes with anti-KCNE3 antibodies. Both KCNE1 and KCNE3 reduced the current density of Kv2.1 and Kv3.1 channels in heterologous expression systems, the mechanism was shown to be via a direct effect on gating kinetics, and not regulating plasma membrane expression [14], as we find for Kv12.2 channels. More recent studies have demonstrated that heterologously expressed Kv4.3 channels, which are activated during neuronal excitation and are believed to have a role in spike repolarization and frequency, are dramatically inhibited by KCNE3 β-subunits by effects on both activation kinetics and current amplitude [40]. We show here for the first time that KCNE subunits may also regulate the activity of a K^+^ channel believed to significantly contribute to neuronal resting potential. Further *in vivo* studies are required to characterize and determine the trafficking dynamics and physiological significance of this Kv12.2 -KCNE1-KCNE3 channel regulatory tripartite complex.

The most pronounced effect of KCNE1 and KCNE3 on Kv12.2 channels is on the regulation of plasma membrane expression. We demonstrate that KCNE1 and/or KCNE3 β-subunits did not affect overall Kv12.2 channel total protein expression. Previous studies have suggested a similar role of the KCNE β-subunits in K^+^ channel regulation. Nicolas *et al*. (2001) demonstrated in KCNE1 knock-out mice, that KCNQ1 channels do not traffic to the apical membranes of vestibular dark cells, where they would normally be found in wild type cells [41], suggesting that KCNE1 β-subunits chaperone KCNQ1 to the membrane surface or alternatively stabilize membrane expression. Interestingly KCNE3 has been shown previously to inhibit hERG channels, suggesting either the formation of a non-functional channel, or one that fails to reach the plasma membrane [29]. Similarly in our study Kv12.2 channel currents were increased by the knockdown of endogenous KCNE1 and/or KCNE3 β-subunits, and attenuated by the over-expression. Our results clearly show that increased Kv12.2 current following knockdown of KCNE1 and KCNE3 occurs primarily through enhanced surface expression. We assume Kv12.2 current reductions induced by KCNE1 and KCNE3 overexpression result from reduced surface expression, although we did not definitively determine the mechanism using the surface biotinylation assay. KCNQ1-KCNE1 assembly is chaperone mediated, suggesting that these proteins first associate in either the ER or cis-Golgi; as KCNQ1 channels assemble early in the secretory pathway [42]. This suggests the possibility that KCNE subunits could affect membrane trafficking of K^+^ channels by control of retention in or export from the ER. Interestingly a recent study by Xu *et al*. (2009), demonstrated that KCNQ1-KCNE1 complexes but not homomeric KCNQ1 channels undergo clathrin- and dynamin 2-dependent internalization; redefining KCNE1 as an endocytic chaperone for KCNQ1 [43]. Further studies are required to define whether KCNE1 and KCNE3 determine membrane surface expression of Kv12.2 through control of export to the plasma membrane, promotion of endocytic recycling, or a combination of both mechanisms.

The subunit composition of KCNE-containing channel complexes can greatly influence channel function [10], and it is therefore important to understand the stoichiometry of native channel complexes. Here we demonstrate *in vivo* that both KCNE1 and KCNE3 appear to simultaneously interact with individual Kv12.2 channel tetramers. Remixing of subunits during the co-IP assays to produce these results remains a possibility, but is extremely unlikely due to high dilution factors and removal of the proteins from the membrane context. Recent *in vitro* investigations with K^+^ channels have proposed that two KCNE peptides interact with the channel tetramer [35], [36]. Several K^+^ channels, including KCNQ1, hERG and Kv2.1 have been shown to interact with multiple distinct KCNE peptides *in vitro* [15], [16], [20], [24], [25], [44]. One recent study even demonstrated that KCNE1 and KCNE4 can simultaneously interact with KCNQ1 channels in COS-M6 cells [45]. However, the question of whether individual channels may contain multiple distinct KCNE proteins *in vivo* has never been examined prior to this study. We present evidence demonstrating that a significant portion of Kv12.2 channels contain both KCNE1 and KCNE3 β subunits *in vivo*. However, we cannot rule out the possibility that Kv12.2 channels also complex separately with either KCNE1 or KCNE3 in the brain.

We observed an additive increase over the effect of either siRNA alone with both KCNE1 and KCNE3 knockdown on Kv12.2 channel plasma membrane expression. Several mechanisms may have meditated this additive effect. Firstly, it is possible that KCNE1 and KCNE3 have independent effects on Kv12.2 trafficking and are not able to substitute for each other. In this case, both subunits would have to be present for maximal inhibition of Kv12.2 channel expression on the membrane. Alternatively, it may be the case that KCNE1 and KCNE3 do have redundant effects on trafficking in *Xenopus* oocytes, and that removal of either may simply reduce the number of channels that contain a KCNE subunit. In support of this view, KCNE subunits have similar structures [10] and are thus likely to interact with K^+^ channel α-subunits in overlapping binding sites. It would be interesting to determine whether all or only a subset of these KCNE binding sites must be occupied to affect trafficking of Kv12.2 channels. The demonstration of heterologous assembly between a K^+^ channel (Kv12.2) and KCNE family regulatory β-subunits, suggests that the promiscuity of K^+^ channel/KCNE interactions observed *in vitro* may reflect complexity in the stoichiometry of channels formed *in vivo*.

In summary, this study demonstrates for the first time that KCNE1 and KCNE3 β-subunits form a tripartite complex with Kv12.2 channels in the brain; and furthermore suggests that regulation of membrane surface expression, and subsequent channel activation as a function of this association.
